# Endocrine-disrupting chemicals in metabolic bone diseases, including osteoporosis

**DOI:** 10.55730/1300-0144.6128

**Published:** 2025-12-26

**Authors:** Betül GÜNDÜZ, Elif KILIÇ KAN, Ayşegül ATMACA

**Affiliations:** Division of Endocrinology and Metabolism, Department of Internal Medicine, Faculty of Medicine, Ondokuz Mayıs University, Samsun, Turkiye

**Keywords:** Endocrine-disrupting chemicals, bone, osteoporosis

## Abstract

Endocrine disruptors are chemical substances widely utilized across various industrial sectors. Due to their structural similarity to natural ligands, they bind to receptors and influence the endocrine system via agonist–antagonist mechanisms. Exposure occurs through the consumption of contaminated food and water, inhalation of polluted air and dust, and dermal contact. Owing to their dynamic remodeling capacity, bones represent potential targets for endocrine-disrupting chemicals. These chemicals can disrupt bone formation, skeletal development, hormonal regulation, and calcium metabolism. Sensitivity to endocrine-disrupting chemicals is greatest during the prenatal and early postnatal periods. This review summarizes the effects of endocrine-disrupting chemicals on bone tissue.

## 1. Introduction

Endocrine-disrupting chemicals (EDCs) are exogenous substances that interfere with endocrine system functions by altering the physiological synthesis, release, transport, binding, and elimination of hormones in multiple ways [1]. Although their primary sources are industrial materials, they can also be present in certain plant-derived products. Naturally occurring EDCs are hydrophilic and can be eliminated from the body more rapidly. Lipophilic compounds accumulate in adipose tissue and exert long-lasting effects. In daily life, EDCs are encountered in numerous products such as cosmetics, plastic toys, food packaging, furniture, and detergents. Human exposure to EDCs occurs through dermal absorption, inhalation, and ingestion of contaminated food. Phytoestrogens are naturally occurring plant estrogens that are hydrophilic in nature and not stored in adipose tissue. Long-term and high-dose exposure is required for its effects to manifest. Polychlorinated biphenyls, phthalates, bisphenol A, diethylstilbestrol, and dioxins are lipophilic compounds that exert prolonged biological effects.

Animal, cellular, and epidemiological studies have demonstrated that EDCs have significant implications for human health. Many EDCs are structurally similar to natural hormones, particularly estrogen. By exerting agonist–antagonist effects on hormone-sensitive cells, EDCs alter the morphology and physiology of the reproductive, developmental, immune, and other organ systems [2–3]. The adverse health effects of EDC exposure vary according to the age at exposure, duration, elapsed time, dosage, sex, and epigenetic factors [4]. Bones are potential targets of EDCs due to their dynamic structure. Endocrine-disrupting chemicals adversely affect bone metabolism by disrupting bone remodeling and calcium homeostasis. Studies have demonstrated adverse effects on EDCs on bone resorption, formation, remodeling, and bone mineral density [5]. This review summarizes the established and potential effects of endocrine-disrupting chemicals on bone tissue.

## 2. Endocrine-disrupting chemicals and bone

Peak bone mass is primarily determined by genetic factors but is also influenced by sex, environmental conditions, and hormonal regulation. Hormones, prostaglandins, and growth factors regulate bone development, resorption, formation, and remodeling. Several endocrine glands—including the thyroid, parathyroid, adrenal cortex, anterior pituitary, and gonads—regulate osteogenesis. Because of their dynamic structure, bones are potential targets for EDCs.

Exposure to EDCs can lead to serious musculoskeletal conditions, including osteopenia, osteoporosis, fractures, and functional impairments. Epidemiological data indicate that the incidence of these conditions has been steadily increasing [6,7]. Osteoporosis is a systemic skeletal disorder characterized by deterioration of bone microarchitecture and loss of bone mass, resulting in increased bone fragility and fracture susceptibility. EDCs may contribute to the development of osteoporosis by altering osteoblastic and osteoclastic activity [8].

The deleterious effects of EDCs on bone are primarily attributed to the inhibition of osteoblast differentiation rather than the stimulation of osteoclast activity. Impaired osteoblast differentiation results in decreased bone mass and compromised bone quality [9]. Although available studies are limited, exposure to per-and polyfluoroalkyl substances (PFAS) has been suggested to decrease 1.25(OH)D activity and increase parathyroid hormone levels [10]. Vitku et al. conducted a study involving 24 postmenopausal women and reported that higher serum levels of bisphenols and parabens were detected in patients with osteoporosis, potentially disrupting calcium–phosphate metabolism [5]. Estrogen exerts an antiresorptive effect on bone tissue by inhibiting osteoclastic bone resorption. Endocrine-disrupting chemicals (EDCs) exhibiting antiestrogenic activity exert more pronounced deleterious effects on bone [11].

In recent years, research on the effects of endocrine-disrupting chemicals on bone and the musculoskeletal system has expanded considerably. These studies have identified distinct effects of various classes of endocrine disruptors on bone tissue.

The table summarizes the most commonly encountered EDCs, their sources of exposure, mechanisms of action, and documented effects on bone tissue.

### 2.1. Bisphenol A (BPA)

Bisphenol A (BPA) is an endocrine-disrupting compound exhibiting estrogenic activity. It is widely used in the production of plastics, epoxy resins, and thermal papers. Human exposure occurs primarily through ingestion of contaminated food, dermal absorption, or inhalation. BPA has a short biological half-life and is primarily eliminated from the body within approximately 24 h [12]. However, because of its extensive use, humans experience continuous low-level exposure.

Interactions between BPA and sex hormones also influence bone metabolism. Although the effects of BPA on bone remain unclear, several studies have investigated this relationship. Suzuki and Hattori, as well as Hwang et al., suggested that BPA may adversely affect bone turnover by suppressing osteoblastic and osteoclastic activities [13]. However, other studies have reported no significant association between BPA exposure and bone mineral density (BMD). Kim et al. analyzed BPA concentrations, biochemical bone markers, and BMD in 51 postmenopausal women with osteoporosis and found no significant correlations [14]. Zhao et al. reported that BPA exposure had a neutral effect on BMD in premenopausal women [15].

In another study by Suzuki et al. on teleost fish, BPA exposure increased plasma calcium concentrations during the first 4 days. However, both calcitonin and calcium concentrations were reduced by the 8th day [16]. In studies by Kim et al. and Otsuka et al. involving pregnant mice, BPA exposure was reported to upregulate renal genes involved in calcium absorption, thereby decreasing plasma calcium concentrations [17,18].

The inconsistent findings across studies may result from simultaneous exposure to multiple environmental chemicals, making it difficult to attribute effects to a single compound. Nevertheless, reducing exposure is advised by avoiding polycarbonate plastics, refraining from heating them at high temperatures, and minimizing the use of canned products.

### 2.2. Phthalates

Phthalates are synthetic compounds widely used worldwide as plasticizers. They are primarily employed to increase the flexibility and elasticity of plastics. Phthalates are commonly found in plastics, food packaging materials, medical devices, and numerous industrial applications. Human exposure occurs via ingestion, inhalation, or dermal absorption. Phthalates can influence bone metabolism by exerting estrogenic, antiestrogenic, or antiandrogenic activities [11].

In a study involving 480 postmenopausal women, the relationship between urinary phthalate metabolites and BMD at the femoral neck and lumbar spine was examined, revealing that different phthalate metabolites may affect BMD depending on body mass index and age [19]. Agas et al. demonstrated increased fetal bone anomalies associated with phthalate exposure [20]. Sabbieti et al. reported that phthalates induce DNA damage in osteoblasts, exert proapoptotic effects, and disrupt the microfilament network [21]. Bhat et al. proposed that exposure to di-(2-ethylhexyl) phthalate reduces collagen and alkaline phosphatase expression, thereby impairing bone formation [22].

Because phthalate metabolites affect bone metabolism through diverse mechanisms and at varying exposure levels, different interactions with bone tissue are expected depending on the metabolite type, exposure duration, and dose.

### 2.3. Dioxins

Dioxins are lipophilic compounds with a biological half-life of approximately 7–9 years and are poorly metabolized in the human body. They adversely affect endocrine metabolism by modulating gene transcription. Their primary sources are by-products of industrial processes, although they may also originate from natural events such as volcanic activity and forest fires.

Dioxins bind to the aryl hydrocarbon receptor (AhR) expressed in osteoblasts and osteoclasts, thereby regulating gene expression involved in bone metabolism. They inhibit osteoblast differentiation and adversely affect BMD and overall skeletal health [23]. Davies et al. reported that early-life exposure to dioxins and their derivatives reduces peak bone mass and increases the risk of osteoporotic fractures during the postmenopausal period [24]. Alveblom et al. investigated two populations residing on the eastern and western coasts of the Baltic Sea, assessing the effects of dioxins and persistent organochlorines found at high concentrations in fatty fish. The study revealed a higher incidence of vertebral fractures in the eastern population [25]. In a study of 12 Japanese children congenitally exposed to polychlorinated biphenyls (PCBs), Miller identified intrauterine growth restriction, widened fontanelles, and cranial and gingival abnormalities [26]. It has been suggested that the adverse skeletal effects of dioxins are dose-dependent, with clinical manifestations being more pronounced following exposure during prenatal and lactation periods [27].

Conversely, Miettinen et al. reported that the adverse effects of intrauterine exposure to 2,3,7,8-tetrachlorodibenzo-p-dioxin (TCDD) on bone density and mineralization were reversible within 1 year [28]. Eskenazi et al., in a cohort study of women residing in the Seveso, Italy, area 30 years after the 1976 industrial explosion, found no significant association between TCDD exposure and bone health [29].

### 2.4. Per- and polyfluoroalkyl substances (PFAS)

PFAS are widely used in clothing, cosmetics, nonstick coatings, and household furniture. They exhibit a long biological half-life, being slowly eliminated from the human body over approximately 4–8 years due to their low metabolic rate. Women appear to be more susceptible to PFAS toxicity than men.

Studies have demonstrated that PFAS compounds accumulate in bone tissue, leading to osteotoxic effects. Perez et al. demonstrated PFAS accumulation in bone tissue using autopsy samples [30]. Thibodeaux et al. reported that pregnant rats and mice exposed to perfluorooctane sulfonic acid (PFOS) developed fetal bone malformations, with these adverse outcomes being dose and serum PFOS level–dependent [31].

Multiple studies have demonstrated that exposure to PFAS is associated with adverse skeletal and endocrine effects, including reduced BMD, increased fracture risk, delayed puberty, premature menopause, decreased serum estradiol levels, and subclinical hyperthyroidism [32,33]. These adverse outcomes are thought to result from PFAS-induced subclinical hyperthyroidism, reduced serum estradiol, interference with vitamin D metabolism, direct binding to hydroxyapatite crystals, and disruption of bone microarchitecture.

### 2.5. Dichlorodiphenyltrichloroethane (DDT)

DDT has been widely used as an agricultural pesticide. Due to its lipophilic nature, DDT rapidly permeates biological tissues and remains stored in adipose tissue for prolonged periods. Because DDT can be transmitted transgenerationally, its adverse effects may manifest in subsequent generations even in the absence of direct exposure [34]. Although DDT has been banned in many countries because of its adverse effects on human health and the environment, it continues to pose a major public health concern in developing regions [35].

The mechanism by which DDT affects bone metabolism remains unclear. It is believed to influence bone metabolism through its proestrogenic and antiandrogenic activities, as well as by altering thyroid hormone levels and mineralocorticoid synthesis [36–38]. Several studies have demonstrated an inverse correlation between DDT and its metabolites and serum vitamin D concentrations [39,40]. The increased incidence of osteoporosis and fractures among women in northern Europe is believed to be associated with contamination of water sources and the frequent consumption of fish and seafood contaminated with DDT [41]. DDT and its metabolites can cross the placental barrier and induce teratogenic and dysmorphogenetic effects [42].

### 2.6. Alkylphenols

Alkylphenols are nonionic synthetic surfactants used in the production of paints, detergents, adhesives, plastics, and pesticides. They are EDCs exhibiting estrogenic activity. Alkylphenols inhibit spermatogenesis and interfere with testosterone synthesis [43]. Hagiwara et al. demonstrated in pregnant mice that alkylphenol exposure disrupts osteoclast development and induces premature ossification in the fetal skeleton [44]. Prenatal and early postnatal exposure to alkylphenols increases osteocalcin synthesis and induces malformations in the bone diaphysis [45].

### 2.7. Diethylstilbestrol (DES)

Diethylstilbestrol is a synthetic, nonsteroidal estrogen analogue with greater biological activity than estradiol. It was the first estrogenic toxic compound shown to cross the placenta and affect the developing fetus. DES has been used in the treatment of ovarian failure, breast cancer, and prostate cancer, as well as for postmenopausal hormone replacement and miscarriage prevention. However, its widespread use has been associated with benign and malignant tumors, endometritis, and neurodevelopmental disorders [46]. The effects of DES exposure may appear after long latency periods and can induce genetic and epigenetic alterations, resulting in transgenerational transmission [47]. Due to its severe adverse effects, the use of DES has been banned in many countries.

Studies have demonstrated that DES alters bone metabolism and skeletal development [48,49]. Children of mothers who used DES during pregnancy have been shown to exhibit increased bone mass and shortened tubular bones. This phenomenon is thought to be associated with delayed postnatal growth and accelerated ossification [50]. Continuous DES exposure results in increased trabecular bone density, hyperplastic changes, and bone tumors such as osteosarcoma [11].

EDCs are ubiquitous in the environment, and concurrent exposure to multiple EDC types can activate diverse signaling pathways, resulting in cumulative and potentially synergistic effects [51].

## 3. Conclusion

Although studies on the effects of EDCs on bone show variable results, evidence from population and in vivo studies indicates that these compounds alter hormonal pathways—particularly following prenatal and early postnatal exposure—leading to significant skeletal outcomes. A better understanding of the pathophysiology underlying EDC-induced bone damage will contribute to the development of more effective preventive measures. Therefore, further large-scale and comprehensive studies are warranted. Recognizing EDC exposure as a modifiable risk factor for osteoporosis and other metabolic bone disorders could have substantial implications for improving bone health.

## Figures and Tables

**Table:** Sources of exposure, mechanisms of action, and effects on bone tissue of endocrine-disrupting chemicals (EDCs).

Endocrine-disrupting chemicals	Sources of exposure	Mechanisms of action	Effects on bone tissue
Bisphenol A (BPA)	Plastics, epoxy resins, thermal papers	Effect sex steroids Suppress osteoclastic and osteoblastic activity	BMD remains unchanged or decreases
Phthalates	Plastics, food packaging, medical devices	Effect sex steroids	Decrease in BMD Fetal bone abnormalities
Dioxins	Industrial by-products	Effect aryl hydrocarbon receptor (AhR)	Decrease in BMD and bone mass Increased risk of osteoporotic fractures
Per- and polyfluoroalkyl Substances (PFAS)	Clothing, cosmetics, nonstick coatings, furniture	Effect sex steroids Effect thyroid hormones Effect vitamin D	Decrease in BMD Increased risk of osteoporotic fractures
Dichlorodiphenyltrichloroethane (DDT)	Pesticide	Effect sex steroids Effect thyroid hormones Effect mineralocorticoid	Dysmorphogenetic effects
Alkylphenols	Nonionic synthetic surfactant used in the production of paints, detergents, adhesives, plastics, and pesticides	Effect sex steroids	Decrease in BMD
Diethylstilbestrol (DES)	Nonsteroidal estrogen analogue	Effect sex steroids	Increased bone mass, shortening of tubular bones

## References

[1] La Merrill MA Vandenberg LN Smith MT Goodson W Browne P Consensus on the key characteristics of endocrine-disrupting chemicals as a basis for hazard identification Nature Reviews Endocrinology 2020 16 1 45 57 10.1038/s41574-019-0273-8 PMC6902641 31719706

[2] Cano-Sancho G Salmon AG La Merrill MA Association between exposure to p,p’-ddt and its metabolite p,p’-dde with obesity: integrated systematic review and meta-analysis Environmental Health Respectives 2017 125 9 096002 10.1289/EHP527 PMC5915185 28934091

[3] Ghassabian A Trasande L Disruption in thyroid signaling pathway: a mechanism for the effect of endocrine-disrupting chemicals on child neurodevelopment Frontiers in Endocrinology 2018 9 204 10.3389/fendo.2018.00204 29760680 PMC5936967

[4] Zoeller RT Brown TR Doan LL Gore AC Skakkebaek NE Endocrine-disrupting chemicals and public health protection: a statement of principles from the endocrine society Endocrinology 2012 153 9 4097 4110 10.1210/en.2012-1422 22733974 PMC3423612

[5] Vitku J Kolatorova L Franekova L Blahos J Simkova M Endocrine disruptors of the bisphenol and paraben families and bone metabolism Physiological Research 2018 67 455 464 10.33549/physiolres.934005 30484672

[6] Lane NE Shidara K Wise BL Osteoarthritis year in review 2016: clinical Osteoarthritis and Cartilage 2017 25 2 209 215 10.1016/j.joca.2016.09.025 28100423

[7] Quevedo I Ormeño JC Weissglas B Opazo C Epidemiology and direct medical cost of osteoporotic hip fracture in Chile Journal of Osteoporosis 2020 2020 5360467 10.1155/2020/5360467 32273971 PMC7132582

[8] Toni R Conza GD Barbaro F Zini N Consolini E Microtopography of immune cells in osteoporosis and bone lesions by endocrine disruptors Frontiers in Immunology 2020 11 1737 10.3389/fimmu.2020.01737 33013826 PMC7493744

[9] Agas D Lacava G Sabbieti MG Bone and bone marrow disruption by endocrine active substances Journal of Cellular Physiology 2018 234 1 192 213 10.1002/jcp.26837 29953590

[10] Leko MB Pleic N Falchetti A Environmental factors that affect parathyroid hormone and calcitonin levels International Journal of Molecular Sciences 2021 23 1 44 10.3390/ijms23010044 35008468 PMC8744774

[11] Yaglova NV Yaglov VV Endocrine Disruptors as a new etiologic factor of bone tissue diseases Sovremennye Tekhnologii v Meditsine 2021 13 2 84 94 10.17691/stm2021.13.2.10 34513081 PMC8353721

[12] Thayer KA Doerge DR Hunt D Schurman SH Twaddle NC Pharmacokinetics of bisphenol a in humans following a single oral administration Environment International 2015 83 107 115 10.1016/j.envint.2015.06.008 26115537 PMC4545316

[13] Hwang JK Min KH Choi KH Hwang YC Jeong K Bisphenol a reduces differentiation and stimulates apoptosis of osteoclasts and osteoblasts Life Sciences 2013 93 9–11 367 372 10.1016/j.lfs.2013.07.020 23900028

[14] Kim DH Oh CH Hwang Y Jeong K Ahn KJ Serum bisphenol a concentration in postmenopausal women with osteoporosis Journal of Bone Metabolism 2012 19 2 87 93 10.11005/jbm.2012.19.2.87 24524038 PMC3780927

[15] Zhao H Bi Y Ma L Zhao L Wang T The effects of bisphenol a (bpa) exposure on fat mass and serum leptin concentrations have no impact on bone mineral densities in non-obese premenopausal women Clinical Biochemistry 2012 45 18 1602 1606 10.1016/j.clinbiochem.2012.08.024 22981830

[16] Suzuki N Kambegawa A Hattori A Bisphenol a influences the plasma calcium level and inhibits calcitonin secretion in goldfish Zoological Science 2003 20 6 745 748 10.2108/zsj.20.745 12832826

[17] Kim S An BS Yang H Jeung EB Effects of octylphenol and bisphenol a on the expression of calcium transport genes in the mouse duodenum and kidney during pregnancy Toxicology 2013 303 99 106 10.1016/j.tox.2012.10.023 23142789

[18] Otsuka H Sugimoto M Ikeda S Kume S Effects of bisphenol a administration to pregnant mice on serum ca and intestinal ca absorption Animal Science Journal 2012 83 3 232 237 10.1111/j.1740-0929.2011.00947.x 22435627

[19] DeFlorio-Barker SA Turyk ME Associations between bone mineral density and urinary phthalate metabolites among postmenopausal women: a cross-sectional study of NHANES data 2005–2010 International Journal of Environmental Health Research 2016 26 3 326 345 10.1080/09603123.2015.1111312 26586408

[20] Agas D Sabbieti MG Marchetti L Endocrine disruptors and bone metabolism Archives of Toxicology 2013 87 4 735 751 10.1007/s00204-012-0988-y 23192238

[21] Sabbieti MG Agas D Santoni G Materazzi S Menghi G Marchetti L Involvement of p53 in phthalate effects on mouse and rat osteoblasts Journal of Cellular Biochemistry 2009 107 2 316 327 10.1002/jcb.22127 19330797

[22] Bhat FA Ramajayam G Parameswari S Vignesh RC Karthikeyan S Di 2-ethyl hexyl phthalate affects differentiation and matrix mineralization of rat calvarial osteoblasts – in vitro Toxicology in Vitro 2013 27 1 250 256 10.1016/j.tiv.2012.09.003 22985736

[23] Watson ATD Nordberg RC Loboa EG Kullman SW Evidence for aryl hydrocarbon receptor-mediated inhibition of osteoblast differentiation in human mesenchymal stem cells Toxicological Sciences: an Official Journal of the Society of Toxicology 2018 167 1 145 146 10.1093/toxsci/kfy225 PMC6317429 30203000

[24] Davies JH Evans BAJ Gregory JW Bone mass acquisition in healthy children Archives of Disease in Childhood 2005 90 4 373 378 10.1136/adc.2004.053553 15781927 PMC1720329

[25] Alveblom AK Rylander L Johnell O Hagmar L Incidence of hospitalized osteoporotic fractures in cohorts with high dietary intake of persistent organochlorine compounds International Archives of Occupational and Environmental Health 2003 76 3 246 248 10.1007/s00420-002-0408-3 12690500

[26] Miller RW Congenital PCB poisoning: a reevaluation Environmental Health Respective 1985 60 211 214 10.1289/ehp.8560211 PMC1568560 3928346

[27] Sholts SB Korkalainen M Simanainen U Miettinen HM Hakansson H *In utero*/lactational and adult exposures to 2,3,7,8-tetrachlorodibenzo-p-dioxin (TCDD) show differential effects on craniofacial development and growth in rats Toxicology 2015 337 30 38 10.1016/j.tox.2015.08.010 26320568

[28] Miettinen HM Pulkkinen P Jämsä T Koistinen J Simanainen U Effects of *in utero* and lactational TCDD exposure on bone development in differentially sensitive rat lines Toxicological Sciences 2005 85 2 1003 1012 10.1093/toxsci/kfi136 15746008

[29] Eskenazi B Warner M Sirtori M Fuerst T Rauch SA Serum dioxin concentrations and bone density and structure in the seveso women’s health study Environmental Health Respectives 2014 122 1 51 57 10.1289/ehp.1306788 PMC3888571 24240199

[30] Pérez F Nadal M Navarro-Ortega A Fábrega F Domingo JL Accumulation of perfluoroalkyl substances in human tissues Environment International 2013 59 354 362 10.1016/j.envint.2013.06.004 23892228

[31] Thibodeaux JR Hanson RG Rogers JM Grey BE Barbee BD Exposure to perfluorooctane sulfonate during pregnancy in rat and mouse. ı: maternal and prenatal evaluations Toxicological Sciences 2003 74 2 369 381 10.1093/toxsci/kfg121 12773773

[32] Chevalley T Bonjour JP van Rietbergen B Rizzoli R Ferrari S Fractures in healthy females followed from childhood to early adulthood are associated with later menarcheal age and with impaired bone microstructure at peak bone mass The Journal of Clinical Endocrinology and Metabolism 2012 97 11 4174 4181 10.1210/jc.2012-2561 22948760

[33] Knox SS Jackson T Javins B Frisbee SJ Shankar A Implications of early menopause in women exposed to perfluorocarbons The Journal of Clinical Endocrinology and Metabolism 2011 96 6 1747 1753 10.1210/jc.2010-2401 21411548 PMC3206400

[34] Kabasenche WP Skinner MK DDT, epigenetic harm, and transgenerational environmental justice Environmental Health: a Global Access Science Source 2014 13 62 10.1186/1476-069X-13-62 25086599 PMC4124473

[35] World Health Organization (WHO) & Food and Agriculture Organization of the United Nations (FAO) Pesticide residues in food 2016 evaluations. Part II Toxicological Geneva WHO and FAO 2017 (WHO Technical Report Series)

[36] Yaglova NV Yaglov VV Cytophysiological changes in the follicular epithelium of the thyroid gland after long-term exposure to low doses of dichlorodiphenyltrichloroethane (DDT) Bulletin of Experimental Biology and Medicine 2017 162 5 699 702 10.1007/s10517-017-3691-4 28361414

[37] Yaglova NV Tsomartova DA Yaglov VV Differences in production of adrenal steroid hormones in pubertal rats exposed to low doses of the endocrine disruptor DDT during prenatal and postnatal development Biomeditsinskaia Khimiia 2017 63 4 306 311 10.18097/PBMC20176304306 28862600

[38] El-Hefnawy T Hernandez C Stabile PA The endocrine disrupting alkylphenols and 4,4′-DDT interfere with estrogen conversion and clearance by mouse liver cytosol Reproductive Biology 2017 17 3 185 192 10.1016/j.repbio.2017.04.003 28532594

[39] Morales E Gascon M Martinez D Casas M Ballester F Associations between blood persistent organic pollutants and 25-hydroxyvitamin D3 in pregnancy Environment International 2013 57–58 34 41 10.1016/j.envint.2013.03.011 23651836

[40] Cianferotti L Bertoldo F Bischoff-Ferrari HA Bruyere O Cooper C Vitamin D supplementation in the prevention and management of major chronic diseases not related to mineral homeostasis in adults: research for evidence and a scientific statement from the European society for clinical and economic aspects of osteoporosis and osteoarthritis (ESCEO) Endocrine 2017 56 2 245 261 10.1007/s12020-017-1290-9 28390010 PMC6776482

[41] Wallin E Rylander L Hagmar L Exposure to persistent organochlorine compounds through fish consumption and the incidence of osteoporotic fractures Scandinavian Journal of Work, Environment and Health 2004 30 1 30 35 10.5271/sjweh.762 15018026

[42] Yaglova NV Obernikhin SS Tsomartova DA Nazimova SV Yaglov VV Expression of transcription factor PRH/Hhex in adrenal chromaffin cells in the postnatal development and its role in the regulation of proliferative processes Bulletin of Experimental Biology and Medicine 2018 165 4 508 511 10.1007/s10517-018-4205-8 30121926

[43] De Falco M Forte M Laforgia V Estrogenic and anti-androgenic endocrine disrupting chemicals and their impact on the male reproductive system Frontiers in Environmental Science 2015 3 10.3389/fenvs.2015.00003 PMC4476199 26157793

[44] Hagiwara H Sugizaki T Tsukamoto Y Senoh E Goto T Effects of alkylphenols on bone metabolism in vivo and in vitro Toxicology Letters 2008 181 1 13 18 10.1016/j.toxlet.2008.06.863 18621119

[45] Kamei S Miyawaki J Sakayama K Yamamoto H Masuno H Perinatal and postnatal exposure to 4-tert-octylphenol inhibits cortical bone growth in width at the diaphysis in female mice Toxicology 2008 252 1–3 99 104 10.1016/j.tox.2008.08.008 18790001

[46] Kioumourtzoglou MA Coull BA O’Reilly EJ Ascherio A Weisskopf MG Association of exposure to diethylstilbestrol during pregnancy with multigenerational neurodevelopmental deficits Jama Pediatrics 2018 172 7 670 677 10.1001/jamapediatrics.2018.0727 29799929 PMC6137513

[47] Newbold RR Padilla-Banks E Jefferson WN Adverse effects of the model environmental estrogen diethylstilbestrol are transmitted to subsequent generations Endocrinology 2006 147 6 11 17 10.1210/en.2005-1164 16690809

[48] Pelch KE Carleton SM Phillips CL Nagel SC Developmental exposure to xenoestrogens at low doses alters femur length and tensile strength in adult mice Biology of Reproduction 2012 86 3 69 10.1095/biolreprod.111.096545 22088916 PMC3316267

[49] Rowas SA Haddad R Gawri R Al Ma’awi AA Chalifour LE Effect of *in utero* exposure to diethylstilbestrol on lumbar and femoral bone, articular cartilage, and the intervertebral disc in male and female adult mice progeny with and without swimming exercise Arthritis Research and Therapy 2012 14 1 R17 10.1186/ar3696 22269139 PMC3392807

[50] Migliaccio S Newbold RR Bullock BC Mclachlan JA Korach KS Developmental exposure to estrogens induces persistent changes in skeletal tissue Endocrinology 1992 130 3 1756 1758 10.1210/endo.130.3.1537323 1537323

[51] Turan S Endocrine disrupting chemicals and bone Best Practice and Research Clinical Endocrinology and Metabolism 2021 35 5 101495 10.1016/j.beem.2021.101495 33618984

